# Tracking arboviruses, their transmission vectors and potential hosts by nanopore sequencing of mosquitoes

**DOI:** 10.1099/mgen.0.001184

**Published:** 2024-01-19

**Authors:** Jeremy D. Mirza, Lilian de Oliveira Guimarães, Sam Wilkinson, Esmenia C. Rocha, Mayara Bertanhe, Vanessa Christe Helfstein, Juliana Telles de-Deus, Ingra M. Claro, Nicola Cumley, Joshua Quick, Nuno R. Faria, Ester C. Sabino, Karin Kirchgatter, Nicholas J. Loman

**Affiliations:** ^1^​ Institute of Inflammation and Ageing, University of Birmingham, Birmingham, UK; ^2^​ Department of Biosciences, University of Birmingham, Birmingham, UK; ^3^​ Instituto Pasteur, São Paulo, Brazil; ^4^​ Instituto de Medicina Tropical, Faculdade de Medicina da Universidade de São Paulo, São Paulo, Brazil; ^5^​ MRC Centre for Global Infectious Disease Analysis, Jameel Institute, Imperial College London, London, UK

**Keywords:** arboviruses, cytochrome c oxidase, metagenomics, mosquitoes, nanopore, SMART-9N

## Abstract

The risk to human health from mosquito-borne viruses such as dengue, chikungunya and yellow fever is increasing due to increased human expansion, deforestation and climate change. To anticipate and predict the spread and transmission of mosquito-borne viruses, a better understanding of the transmission cycle in mosquito populations is needed. We present a pathogen-agnostic combined sequencing protocol for identifying vectors, viral pathogens and their hosts or reservoirs using portable Oxford Nanopore sequencing. Using mosquitoes collected in São Paulo, Brazil, we extracted RNA for virus identification and DNA for blood meal and mosquito identification. Mosquitoes and blood meals were identified by comparing cytochrome c oxidase I (COI) sequences against a curated Barcode of Life Data System (BOLD). Viruses were identified using the SMART-9N protocol, which allows amplified DNA to be prepared with native barcoding for nanopore sequencing. Kraken 2 was employed to detect viral pathogens and Minimap2 and BOLD identified the contents of the blood meal. Due to the high similarity of some species, mosquito identification was conducted using blast after generation of consensus COI sequences using RACON polishing. This protocol can simultaneously uncover viral diversity, mosquito species and mosquito feeding habits. It also has the potential to increase understanding of mosquito genetic diversity and transmission dynamics of zoonotic mosquito-borne viruses.

## Abbreviations

BOLD, barcode of life database; CDC, Centers for Disease Control; CFS, Coordenadoria de Fauna Silvestre; CHIKV, chikungunya; COI, cytochrome c oxidase I; CxFV, Culex flavivirus; DENV, dengue; ENA, European Nucleotide Archive; FAPESP, Fundação de Amparo à Pesquisa do Estado de São Paulo; HAC, high accuracy basecalling; H-V-P, host-vector-pathogen; NFW, nuclease-free water; ONT, Oxford Nanopore Technologies; PCR, polymerase chain reaction; PEFI, Parque Estadual das Fontes do Ipiranga; SMART-9N, Switching Mechanism at the 5’ end; TSO, template switching oligo; YFV, yellow fever; ZIKV, Zika.

## Impact Statement

This research presents a rapid means of identifying RNA viruses directly (without culture) from mosquitoes whilst simultaneously identifying the mosquito species and its host through blood meal identification. This combination of information presents an opportunity to link novel arboviruses to the mosquito vectors of these pathogens. Further, it allows for the identification of animals that can potentially be infected by these viruses, identifying whether there is a risk of spillover into human populations. This species-agnostic method is combined with portable nanopore sequencing, permitting discovery of novel arboviruses in remote environments. Finally, this method can form the basis of an early warning detection system by identifying arboviruses before spillovers into human populations, providing a system for pre-empting future arboviral epidemics.

## Data Summary

fastq files for amplicon and metagenomic sequences generated using nanopore sequencing for these experiments have been deposited and made publicly available in the European Nucleotide Archive (ENA) under the project accession number PRJEB67345, Study accession ID ERP152382. fastq files are available at ENA under accession numbers ERX11591481–ERX11591610. Barcodes, accession numbers and associated metadata are listed in the Supplementary Tables (Tables S1 and S2, available in the online version of this article).

## Introduction

Urbanization, climate change and human expansion have led to an increased risk of mosquito-borne viruses reaching human populations. This risk of zoonotic disease transfer via a mosquito-derived vector is rising, as shown by the continuous increase in the emergence of zoonotic diseases for over half a century [1, 2]. Recent epidemics caused by Zika (ZIKV; *Flavivirus*; *Flaviviridae*), dengue (DENV; *Flavivirus*; *Flaviviridae*), yellow fever (YFV; *Flavivirus*; *Flaviviridae*) and chikungunya (CHIKV; *Alphavirus*; *Togaviridae*) viruses transmitted by *Aedes aegypti* and *Aedes albopictus* mosquitoes led to several million human infections, thousands of deaths and large socio-economic losses [3, 4].

The detection of mosquito-borne viruses can be limited by the lack of molecular protocols, reliance on syndromic surveillance of human febrile cases and co-circulation of viruses that cause similar clinical symptoms [5]. For example, ZIKV circulated undetected for over 1 year in Brazil [6, 7] before the first cases were reported in 2015 in northeast Brazil [8, 9]. Once identified, ZIKV infections were then associated with microcephaly in newborns [10]. Autochthonous transmission was reported in 87 countries and territories in both tropical and subtropical regions [11], with cases in more temperate regions such as Florida and Texas in the USA [12, 13] and Marseille in France [14].

Implementing routine virus surveillance in mosquitoes in at-risk areas could help to reduce periods of undetected circulation of mosquito-borne viruses and improve understanding of sylvatic transmission. A case in point is the recent YFV outbreak in Brazil, where *Haemagogus leucocelaenus* and *Haemagogus janthinomys* mosquitoes that feed upon non-human primates and birds at the tree canopy level but can also feed in humans at ground level in deforested habitats [15] were identified as the main mosquito vector species [16]. Unlike vectors such as *Ae. aegypti*, *Haemagogus* spp. do not habit in highly urbanized locations. Molecular detection of YFV in *Haemagogus* spp. and absence of YFV detection in *Aedes* spp. provided evidence for sylvatic but not urban transmission during this large epidemic [16].

Virus surveillance in mosquito populations can also detect rare or novel mosquito-borne viruses either directly in the mosquito populations or in their hosts or reservoirs through analyses of their blood meals. Using a similar pathogen-agnostic sequencing approach, two fatal YFV-suspected cases were found to be caused by Sabia virus, despite testing negative using standard polymerase chain reaction (PCR) detection methods [17]. Moreover, surveillance of viral pathogens in mosquito blood meals offers a promising non-invasive tool to indirectly screen viruses in human populations [18], with potential applications to surveillance of zoonotic pathogens circulating in non-human primates, rodents and birds.

Recent developments in real-time nanopore sequencing allow the rapid amplification and genome sequencing of any viral pathogen without requiring *a priori* information concerning the virus present in a sample [19]. In particular, the recently developed SMART-9N (Switching Mechanism at the 5′ end of RNA Template) metagenomic low-cost protocol (USD $55/sample) allows for the generation of long reads of DNA of up to 18.5 kb [19]. This can cover the complete genome of most mosquito-borne viruses of public health concern, including those from the families *Flaviviridae* (11 kb), *Togaviridae* (10–12 kb) and *Bunyaviridae* (11–19 kb). By combining metagenomic sequencing approaches with powerful *de novo* assembly tools such as Flye, the identification of previously unknown viral species can also be accomplished [20]. This means that even in cases where the virus is not initially characterized, it is now possible to gain comprehensive insights into the viral diversity present in a given sample.

DNA barcoding is a widely employed technique that relies on a conserved DNA region such as the mitochondrial cytochrome c oxidase I gene (COI) to differentiate eukaryotic DNA obtained from mosquitoes and their blood sources [21, 22]. The COI DNA fragment can be amplified via PCR and identified using numerous methods, such as Sanger sequencing, quantitative PCR and next-generation sequencing approaches [23–25]. COI is a valuable marker for eukaryotic barcoding, and the well-established Barcode of Life Data System (BOLD) public database facilitates the comparison of obtained COI sequences with over 1 million recorded entries [26]. Notably, several studies have generated primers capable of identifying mosquito blood meals, covering a wide variety of animals, including birds, mammals, amphibians and reptiles [27].

Previous approaches to identify vectors, viral pathogens and their hosts or reservoirs have been limited in scale, given the cost and complexity of sequencing approaches [25]. Real-time low-cost sequencing using Oxford Nanopore Technologies (ONT) nanopore sequencing has opened up new possibilities for population-level mosquito-borne virus surveillance. Utilizing ONT platforms enables the integration of amplicon-based DNA barcoding with metagenomic sequencing protocols to identify endemic, epidemic, or novel viral pathogens circulating in mosquito populations. This study presents a single combined assay for blood meal, mosquito vector and viral pathogen identification using nanopore sequencing. The developed protocol is tailored to portable nanopore sequencing, making it applicable in remote areas, and promoting deeper insights into host–vector–pathogen dynamics, particularly in poorly characterized mosquito-borne viruses.

## Methods

### Mosquito collection, preparation and taxonomic identification

Mosquitoes were collected at different sites around the zoo of the Coordenadoria de Fauna Silvestre (CFS), situated in the Parque Estadual das Fontes do Ipiranga (PEFI), a public state park with an area of 476 ha in the southeast of São Paulo municipality, São Paulo state, Brazil. Mosquito collections were performed on 2 days a month apart (3 November 2020 and 1 December 2020) at five sites designated by their proximity to points of interest. Collections were performed using automatic traps equipped with either UV or white light (Centers for Disease Control – CDC – miniature light traps) [28] baited with CO_2_ (dry ice), placed in the ground strata (1.5 m off the ground) or canopy (6 to 10 m off the ground). The traps were set at dusk and removed ~14 h later on the following morning. A total of 32 female mosquitoes with engorged abdomens were collected and identified, separated and then frozen in the field using liquid nitrogen. Subsequently, the specimens were transported to Instituto Pasteur, São Paulo, where they underwent identification using taxonomic keys [29–31].

### Primer selection

Primer schemes from previous publications were tested for both the blood meal and mosquito COI regions [24, 27]. Two sets of primers for the identification of blood meals (COI long and COI short forward and reverse) were identified, tested and used for PCR amplicon generation [27]. Two sets of pairs of primers for the identification of mosquito species were also tested (Anoph and Mos forward and reverse) and used to identify COI corresponding to the vector species [24]. Primers for virus metagenomic sequencing were adapted from Claro and colleagues [19]. These consist of an oligomer consisting of every base combination for nine positions, along with a complementary template switching oligo (TSO) for cDNA formation.

### DNA preparation for COI barcoding

Mosquito abdomens were separated and placed in ZR BashingBead Lysis tubes with 400 µl of DNA/RNA shield (Cambridge Bioscience) and placed in a bead beater homogenizer for five rounds of 300 r.p.m. for 1 min each, being placed on ice between each round. Following this, samples were centrifuged at 10 000 ***g*** for 5 min and the pellet was discarded. DNA was extracted using the Quick-DNA kit and its protocol (Zymo Research) and eluted into 30 µl nuclease-free water (NFW) for each sample. A PCR reaction was set up in three separate tubes with 2.5 µl of DNA added for each sample, to a reaction volume of 25 µl, with 12.5 µl Q5 High-Fidelity 2× Master Mix, 4 µl of each COI primer pool at 10 µM and 6 µl of NFW. The blood meal primers [27] were run in two wells, separating the COI_long and COI_short primers. After heat activation at 95 °C for 60 s, PCR was run for 35 cycles, with denaturation at 95 °C for 30 s, annealing at 62 °C for 50 s and an extension at 72 °C for 60 s. Both sets of mosquito primers [24] were run in the same well, with an initial heat activation at 95 °C for 60 s, followed by 35 cycles of denaturing at 95 °C for 30 s, annealing at 56 °C for 20 s and an extension at 72 °C for 30 s. After PCR, amplicons were pooled for each mosquito sample and quantified using the Qubit dsDNA High Sensitivity assay (cat no. Q32854, Life Technologies, USA) on the Qubit 3.0 instrument (Life Technologies, USA). For each amplicon pool, 10–20 ng of DNA was reserved for sequencing library preparation.

### RNA preparation for virus metagenomics

Mosquito head regions were separated and placed in ZR BashingBead Lysis tubes with 400 µl of DNA/RNA shield and placed in a bead beater homogenizer for five rounds of 300 r.p.m. for 1 min each. Following this, samples were centrifuged at 10 000 ***g*** for 5 min and the pellet was discarded. RNA was extracted using the Quick-RNA kit (Zymo Research) and eluted into 50 µl. Extracted RNA was treated using TURBO DNase (cat no. AM2239, Thermo Fisher Scientific, USA) at 37 °C for 30 min and then concentrated to 10 µl using the Zymo RNA clean and concentrator-5 (Zymo Research). SMART cDNA synthesis and PCR was performed using a previously described protocol [19] with the NEB-PCR oligo for amplification. Following PCR, amplicon DNA was quantified using a Qubit dsDNA High Sensitivity assay (cat no. Q32854, Life Technologies, USA), with 100–150 ng being reserved for sequencing library preparation.

### Nanopore sequencing

To combine COI DNA pools and SMART-9N amplicon pools for each mosquito, an approximate DNA concentration ratio of 1 : 10 was used. MinION libraries were prepared using 50 ng of each DNA pool, and barcoding was carried out using the EXP-NBD114.96 Native barcoding kits to allow for multiplexing of up to 96 samples. Sequencing libraries were prepared using the SQK-LSK109 kit and the standard nanopore protocol for multiplex sequencing [9, 32]. R9.4.1 MinION flow cells were prepared to run 10–12 samples at a time and were run on a GridION for 36 h using MinKNOW (23.04.3). Raw FAST5 reads were basecalled and demultiplexed using a high accuracy basecalling model (HAC) with Guppy (6.5.7) to generate fastq files for each barcoded sample.

### Database curation for mosquito and blood meal identification

COI identification was performed using a manually created database extracted from BOLD [26]. Databases accessed and used were obtained on 20 November 2022. For blood meal identification, all global mammalian, avian, amphibian and reptilian sequences were downloaded, and for the mosquito identification all global Culicidae references were downloaded, both as multifasta files. The collated database repositories were then parsed using a Python script (https://github.com/BioWilko/co1_fish/blob/0.1/co1_fish/ref_parser.py) to filter out duplicate reads for the same species, selecting the COI sequence with the lowest N count and largest coverage, while omitting reads with significant divergence from COI.

### Mosquito identification pipeline

fastq files were mapped against the Culicidae COI database using co1_fish (https://github.com/BioWilko/co1_fish/tree/0.1) as a wrapper for Minimap2 [33]. Reads mapping against the mosquito database were extracted from the fastq files and binned separately. Following this, binned reads were polished using RACON [34], and a generated consensus sequence was compared to a GenBank database using blast [35] to identify the closest matching species for each mosquito.

### Blood meal identification pipeline

fastq files were mapped against the global vertebrate COI database using co1_fish (https://github.com/BioWilko/co1_fish/tree/0.1) as a wrapper for Minimap2 [33] to generate a list of the most probable species in the blood meal. The database entry with the highest number mapped reads when weighted for quality was identified, and any reads that did not map to this were binned separately. Unmapped reads were then rerun with co1_fish to identify any other database hits with a high number of mapped reads.

### Virus identification

Metagenomic sequencing of viruses was performed using Kraken 2, Viral database (RefSeq Viral) [36], generating reports detailing the viruses present in each sample. Identified viruses were aligned against their listed reference sequences in the Kraken 2 report, to generate a SAM file with Minimap2. The SAM file for each viral hit was then sorted, indexed and used to generate a VCF file, which was then used to generate a consensus sequence for each identified virus. The percentage N count based on coverage compared to the reference genome for each virus was assessed, and if *N*<50 %, it was considered that the virus was sufficiently present for identification and inclusion in host–vector pathogen analysis.

## Results

### Overview and costs for the protocol

**Fig. 1.**
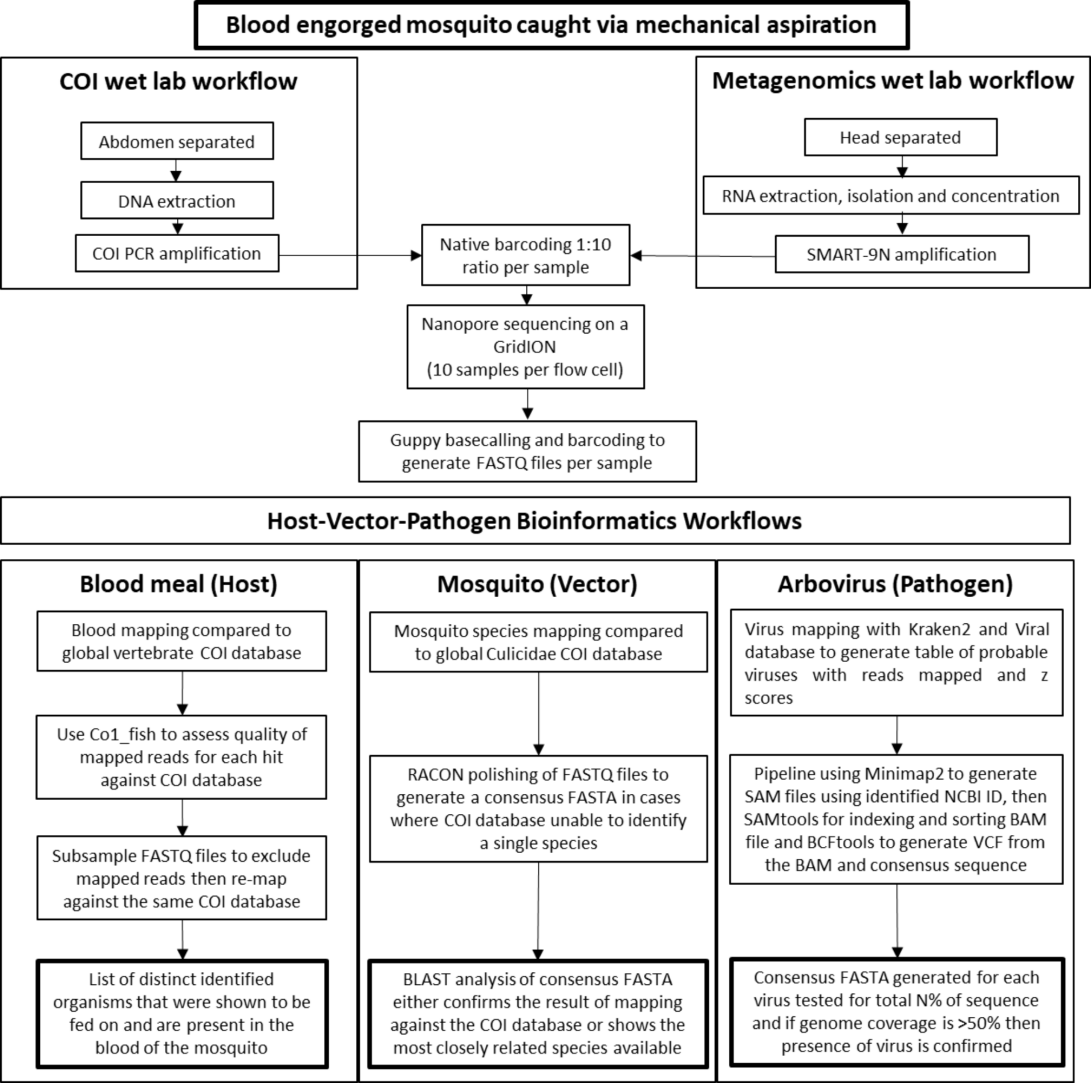
Workflow diagram illustrating the procedure for sequencing and analysing amplicons generated from blood-engorged mosquito abdomens and metagenomic sequencing for virus identification.

The workflow for the combined mosquito–host–virus assay is illustrated in Fig. 1, showcasing the step-by-step process for this comprehensive method. Mosquitoes were caught and females with engorged abdomens, indicating recent blood-feeding, were identified. Abdomens were separated for DNA extraction, while RNA was only extracted from the head region. These were processed following the described methods and loaded onto MinION flow cells in a 1 : 10 ratio (ng µl^−1^), which was sufficient to allow for COI amplicons to be detected, whilst the majority of reads obtained were from the SMART-9N amplicons, allowing for deep metagenomic sequencing. Up to 10 mosquito samples were loaded on each flow cell to allow for sufficient coverage for identification. In total, the full protocol from sample collection to data analysis took 72 h, primarily for in-depth metagenomic sequencing. Virus identification from metagenomic data took around 36 h of sequencing to generate sufficient depth of coverage to identify low-read-count viral pathogens. On the other hand, it was possible to start identifying blood meals and mosquito species in as little as 2 h after initiating sequencing. The median read length (N50) for these sequencing libraries ranged from 1 to 1.1 kb for metagenomic libraries, which was similar to previous studies [19] and sufficient to allow for viral identification, despite a low depth of coverage for some viruses.

Overall, the cost of the protocol from extraction through to sequencing to identify the host, vector and viruses per mosquito sample is approximately UK £48 (USD $61) given the current enzymes and barcoding kits available, considering 10 samples per flow cell. When running the COI amplicons separately it is possible to have up to 30 samples on a cheaper Flongle flow cell, which can reduce costs further. Ultimately, 80 % of the costs are due to the MinION flow cell, combined with the requirement for deep sequencing for viral identification. Additionally, due to the depth and duration of sequencing required for virus identification, recovering sufficient pores from flow cells after running and washing is not yet feasible. Greater depth can be attained running fewer mosquitoes per flow cell. Running the COI barcoding assay on its own can be scaled up to 96 samples, including controls, per MinION flow cell without loss of sample coverage.

### Collection sites and areas for this study

**Fig. 2.**
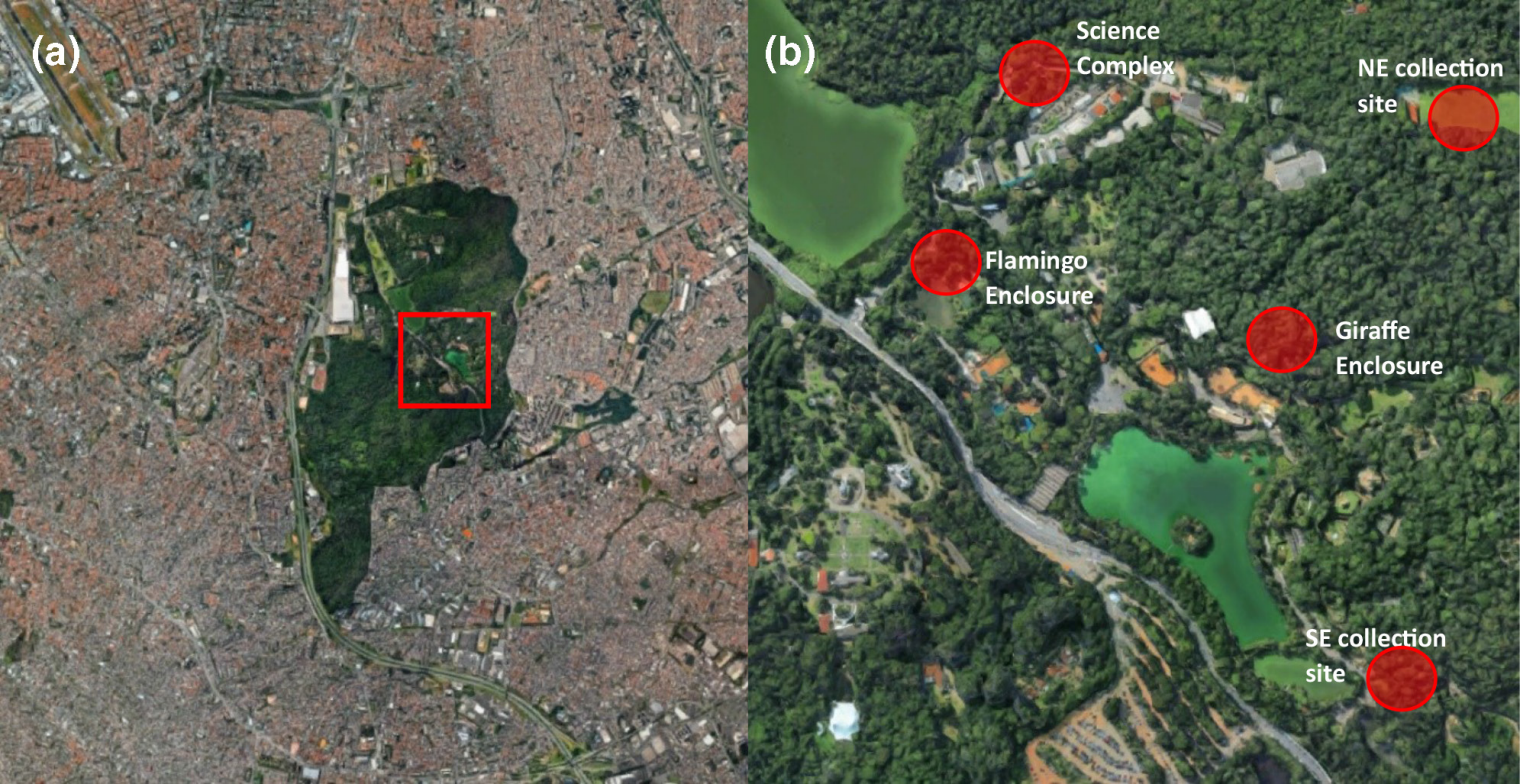
(a) Collection sites and location of Parque Estadual das Fontes do Ipiranga (PEFI) in Sao Paulo, Brazil. (**b**) Sample sites where all mosquitoes were collected from in PEFI. Blood-engorged females identified from each site used in this study are detailed further in Table S2. Collections took place north of the Science Complex, next to the flamingo enclosure, west of the giraffe enclosure and at locations in the south-east and north-east of PEFI that were all used as collection sites in the previous 2015 collection by Guimarães and colleagues [37].

To validate this protocol, collections of mosquitoes were performed at the same sites as in a previous study [37] and results were compared against previous findings. Across multiple collection sites over 2 days it was possible to capture over 30 mosquitoes that had recently fed in a number of locations within the zoo of the CFS, which is located in the Parque Estadual das Fontes do Ipiranga (PEFI) in São Paulo, Brazil (Table S2). While mosquitoes were collected from all sites, higher numbers of blood-engorged females were found in some compared to others. The five sites collected from in this study are spread across different areas in PEFI (Fig. 2). One site is in the northern part of PEFI, above the Science and Research complex, and another is located in the south-east and is the same as a collection site used in the 2015 collection. Two other collection sites are located more centrally in PEFI near to animal enclosures where there is more human activity. These are located next to the flamingo enclosure and to the west of the giraffe enclosure, respectively.

### Validation of the DNA barcoding pipeline

The workflow for blood meal and mosquito analysis was validated using samples already tested in a previous study that were collected at the same site [37]. Mosquitoes had previously been collected in March of 2015, with blood-engorged females being identified taxonomically and processed, and blood meals were identified using Sanger sequencing of the COI regions, with identified sequences being compared against the GenBank database using blast [35]. The extracted DNA samples had been stored for 6 years at −80 °C and were run on the new assay to ensure that the protocol was generating appropriate results. Any discrepancies between the results were then investigated to see if they were caused by sequencing methods, the databases used or potentially sample deterioration over time.

**Fig. 3.**
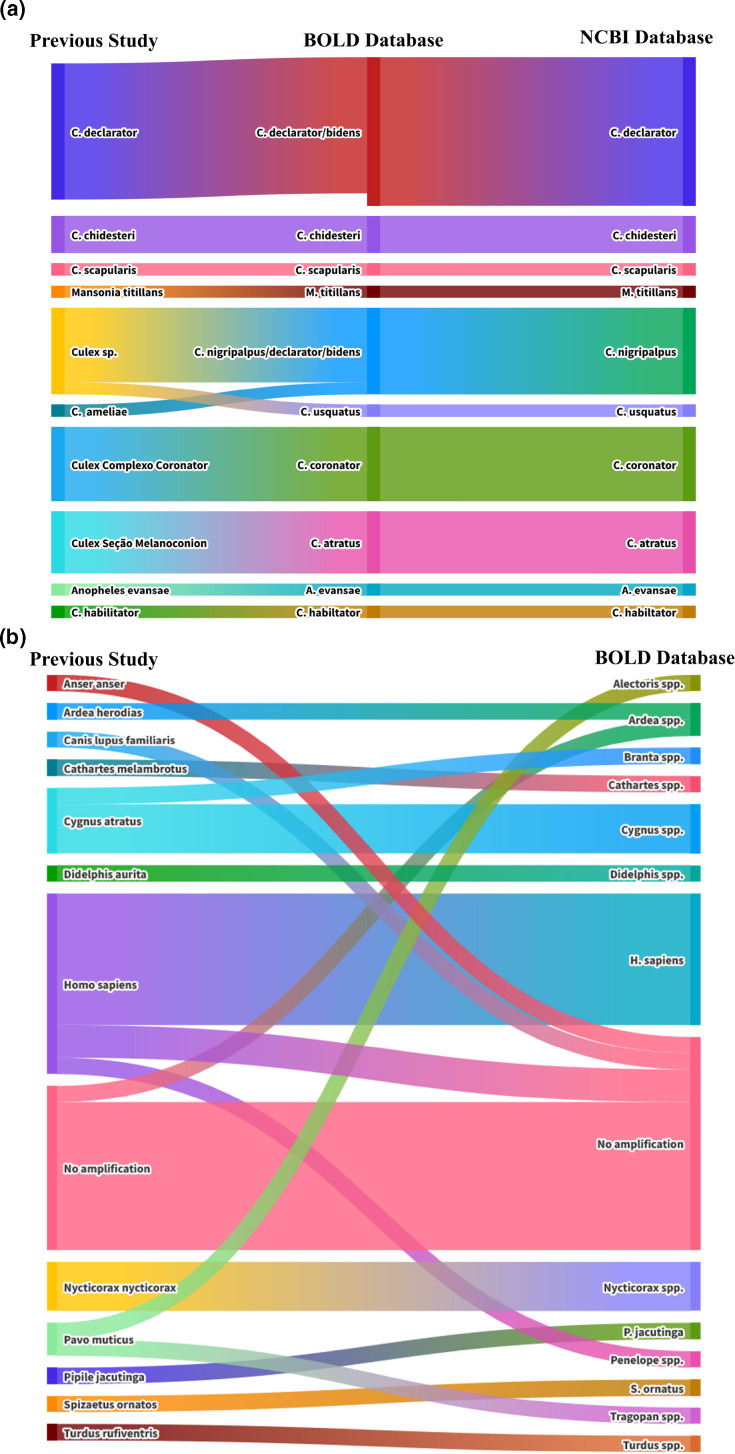
Alluvial diagram showing the identified mosquitoes and blood meals, (**a**) Comparison of results from previous mosquito identification study [37] with the current experiments using BOLD reference and the NCBI database following RACON polishing. (b) Comparison of previous blood meal analysis with the current study using the BOLD global reference database for COI.

For the mosquitoes, it was not possible to separate multiple species identified from the database just using BOLD (Fig. 3a). Partly this was caused by a lack of references for Culicidae specifically recorded from Brazil. To address this limitation, a global Culicidae database was used, including species not previously documented in Latin America. To ensure the reliability of the database, additional manual curation was required, including removing sequences obtained from BOLD that did not map to COI and appeared to be a different gene altogether. Species with multiple entries in the database, such as *Aedes aegypti* and *Culex quinquefasciatus*, were concatenated to reduce bias and weighting over less represented genomes. Finally, sequences that were of a lower quality in terms of the amount of the COI reference covered or had a lack of taxonomic identification below genus level were also removed.

Using this curated BOLD database, it was possible to identify a large number of species from the previous study [37]. For genera other than *Culex*, such as *Aedes*, *Mansonia* and *Anopheles*, the same species was identified as before using nanopore sequencing. However, for some species of *Culex*, such as *C. declarator*, significant reads mapped to multiple genomes, specifically to *C. declarator* and *C. bidens*. For low-diversity sequences, RACON polishing was performed using the first read containing the primer sequence. This was compared against publicly available sequences in blast, allowing for more accurate identification of the corresponding mosquito species. With this it was possible to identify correct species matching the original study, and to further identify mosquitoes that had only been identified by their genus to the species level of the most closely related taxa. Minor discrepancies were observed, in that *C. ameliae* was not found in either BOLD or GenBank, and mosquitoes belonging to the species complex, *Culex* subgenus *Melanoconion*, were identified as *C. atratus*, which is one of the more common species within this group that has been identified and characterized in Brazil [38].

For the blood meal identification it was necessary to use a global vertebrates database from COI, given the general lack of representation of species from Brazil. Given the much greater diversity between all vertebrate species when compared to the Culicidae, it was possible to identify blood meals utilizing the curated BOLD database on its own. Compared to the previous study, previously identified blood meals had no amplification, though a probable cause would be sample degradation over time. Additionally, samples that previously did not have amplification again did not amplify, potentially due to the digested blood meal DNA already being degraded from the beginning (Fig. 3b).

### Observed depth of coverage and outputs from COI amplicon and virome sequencing

**Fig. 4.**
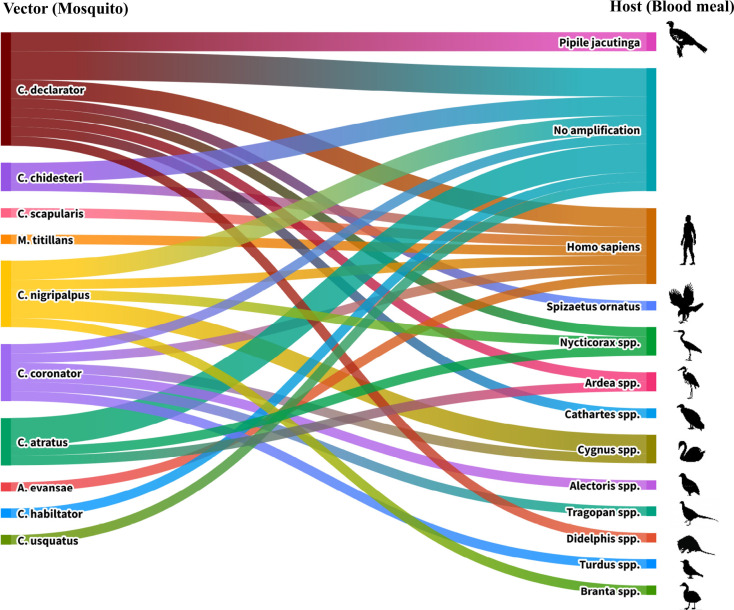
Comparison of host (blood meal) and vector (mosquito) interactions for samples from previous study, using the new identification methods.

The COI primer scheme used (Table 1) has made it possible to obtain high depths of coverage for the amplified regions of COI following nanopore sequencing. Prior to sequencing, the amplified COI regions for the blood meal and mosquito primers were run on a 2 % agarose gel stained with SYBR Gold. From this a large amount of degradation was observed in some of the blood meal samples, likely due to DNA degradation during digestion. Despite this, it was possible to map with over 1000-fold coverage for identified blood meals for some of the mosquitoes collected and, in some cases, identify multiple sources for the blood meal. By using two separate lengths of amplicon for the blood meal primers with degenerate base positions, it has been possible to identify numerous species of birds and mammals (Fig. 4).

**Table 1.** Primers used for host, vector and pathogen identification

Target	Primer name	Sequence	Amplicon size (bp)	Total COI coverage (bp)	Ref.
Host	COI_long (f)	AACCACAAAGACATTGGCAC	663	663	[27]
	COI_long (r)	AAGAATCAGAATARGTGTTG			
	COI_short (f)	GCAGGAACAGGWTGAACCG	324		[27]
	COI_short (r)	AATCAGAAYAGGTGTTGGTATAG			
Vector	Anoph (f)	GCAGGAATTTCTTCTATTTTAGG	275	560	[24]
	Anoph (r)	TAAACTTCAGGGTGACCAAAAAATCA			
	Mos (f)	ATAGTWATACCTATYATAATTGG	299		[24]
	Mos (r)	ACWGTAGTAATAAAATTTACTGC			
Pathogen	SMART-9N	AAGCAGTGGTATCAACGCAGAGTACNNNNNNNNN	–	–	[19]
	TSO	GCTAATCATTGCAAGCAGTGGTATCAACGCAGAGTACATrGrGrG			

F, forward; R, reverse; TSO, template switching oligo.

For the mosquito COI primers, it was possible to run them in the same well despite being in an overlapping region of COI, allowing for simpler PCR and library preparation. For both mosquito and blood meal primers, the depth of coverage for each amplicon varied with different species as well as the level of blood meal digestion. It is possible this is due to some species being amplified more effectively with this primer scheme, although additional factors, such as the size of the blood meal, make this difficult to assess in isolation. Other primers were tested to increase the length of the COI reference gene covered by the amplicons, but these primers provided the best overall combination in terms of the range of species covered, in addition to being relatively convenient for large-scale surveillance.

**Fig. 5.**
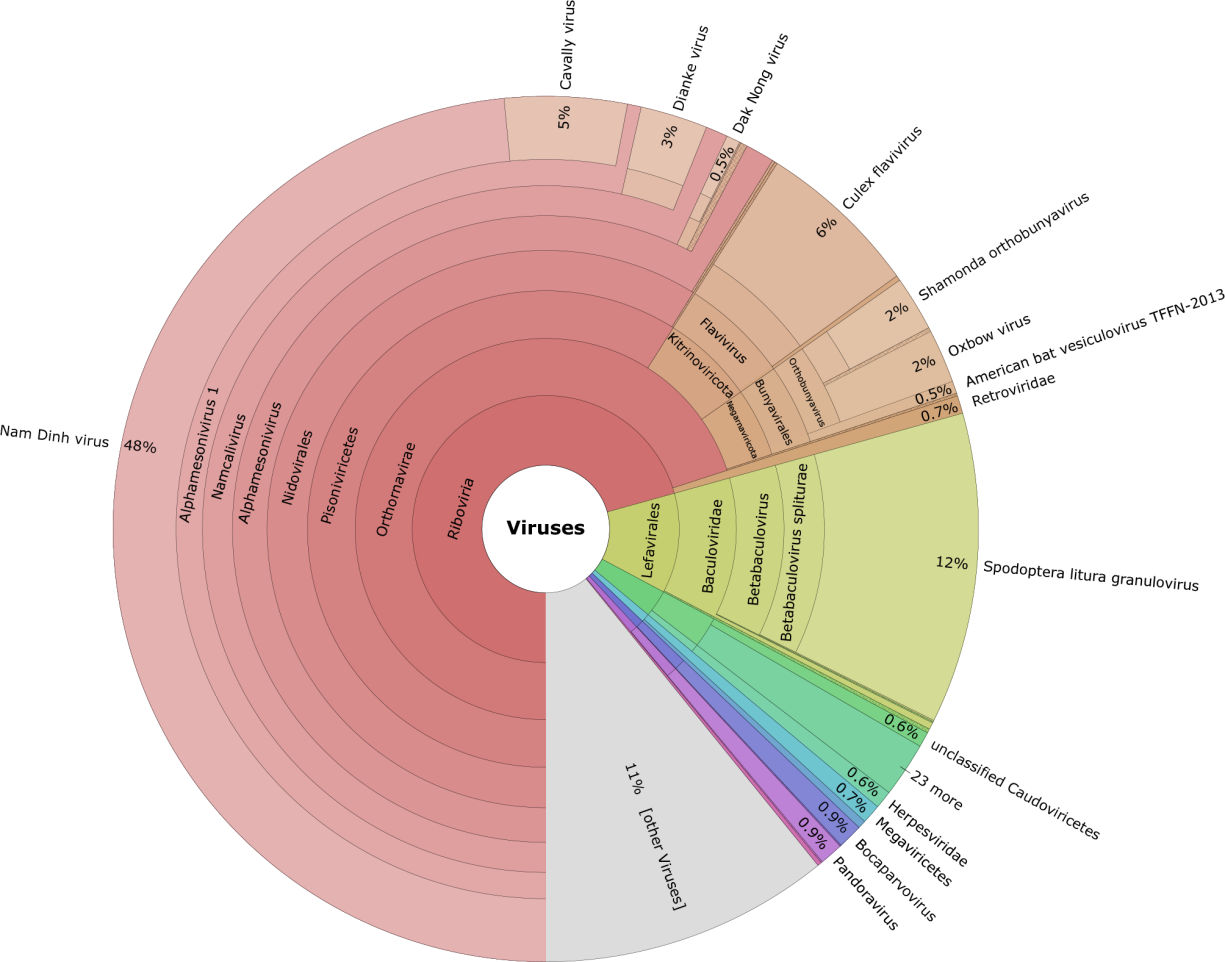
Krona plot illustrating the collective virome present across all blood-engorged mosquito samples.

In addition to extracting DNA from abdomens, RNA was extracted from the head regions separately for each mosquito. Only testing of the head region provided a systemic marker of infection that is much easier than individual salivary gland dissection, and still allowed for the identification of active infections. By collating the viruses identified from sequencing across all samples, it is possible to have a profile of the collective virome for mosquitoes in this area over a given period of time (Fig. 5). Overall, the virome represents <1 % of the total reads captured when sequencing all of the blood-engorged mosquitoes, given that the mass majority of the RNA sequenced belongs to the host as well as bacterial and fungal microbes (97–99 % of total reads across all samples). It is possible that sampling greater numbers of engorged mosquitoes will reveal more active viral infections, causing the relative number of viral reads per sample to increase. At least four different arboviruses are observed with >100 reads in at least 1 sample, including the *Culex* flavivirus (CxFV), Nam Dinh virus, Shamonda orthobunyavirus and *Spodoptera litura* granulovirus.

### Links between hosts and vectors

**Fig. 6.**
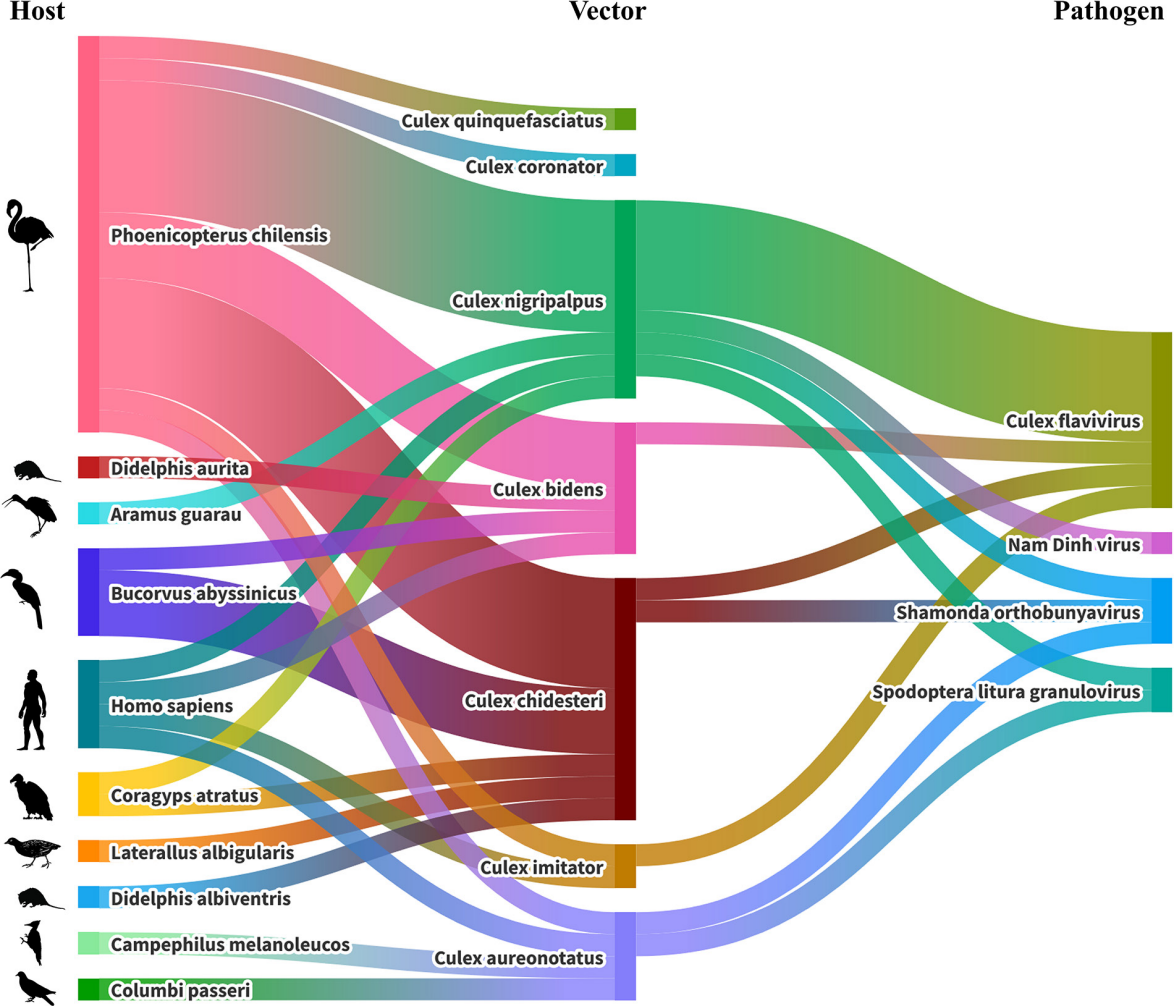
Alluvial flow diagram showing host–vector–pathogen interactions, suggesting possible transmission routes for arboviruses from one host to another by mosquito feeding using identified arboviruses found to be in sufficiently high quantities in terms of genome coverage.

A Sankey plot (Fig. 6) shows the correlations between the different species identified for the blood meal and the mosquito species. All of the mosquito species identified taxonomically or by sequencing were shown to belong to the genus *Culex*, and all blood meals identified were either avian or mammalian in origin, with the majority belonging to birds. Fifteen of the blood meals identified belonged to flamingos that are part of the zoo’s collection of animals. Other zoo attractions, such as the Abyssinian ground hornbill, were identified in four blood meals, as well as a number of wild bird species, such as crakes, vultures and limpkins. Mammalian blood meals identified included four human samples as well as a couple of wild animals.

In terms of feeding dynamics between the hosts and vectors, no mosquito species identified showed a specific feeding behaviour for one host. *C. chidesteri, C. bidens* and *C. nigripalpus* were shown to feed on multiple animals, with every species of *Culex* identified shown to feed on flamingos. *C. chidesteri* was not shown to feed on humans, though given the sample sizes for this it is not currently possible to infer any notion of host selectivity for the different mosquito species.

### Observed host–vector–pathogen (H–V–P) interactions

Of the 32 blood-engorged females sequenced, 12 were shown to have at least 1 RNA virus present. Notably, this included seven *Culex* flaviviruses found in three species of *Culex*. In addition to these flaviviruses, Nam Dinh virus was observed in one individual *C. nigripalpus* mosquito, with over 9500 reads mapping to the reference genome, generating an almost complete consensus sequence at 10× coverage. Other viruses identified included the Shamonda virus, which belongs to the genus Orthobunyavirus and was found in two separate species of *Culex*. Identification of viruses in combination with possible host and vector species can allow for the identification of potential interhost pathways of mosquito-borne viral pathogens.

## Discussion

### General observations of transmission dynamics

From the results it is observed that the majority of blood meals belonged to flamingos that were fed on by six species of *Culex*, including *C. nigripalpus*. Moreover, within this group at least eight arboviruses were identified to have infected the mosquitoes, including Nam Dinh virus. *C. nigripalpus* was also shown to feed on black vultures in this environment. Therefore, even with a small sample size it is possible to conceive how arbovirus infections can be transferred, in this hypothetical case from one avian host to another. However, given the close proximity of humans in environments such as zoos, as well as the lack of host specificity when it comes to mosquitoes’ feeding, it is entirely feasible that one of the identified arboviruses could be transferred to humans [2, 3].

*Spodoptera litura* granulovirus is from the family *Baculoviridae*, which have been shown to infect insect larvae, sawflies and mosquitoes, although currently they have not been shown to be capable of replication in mammalian cells [39, 40]. The Nam Dinh virus is an insect-infecting nidovirus first characterized in 2011 from mosquitoes in Vietnam [41]. Subsequently this has been identified in other parts of Southeast Asia, such as in *C. pipiens quinquefasciatus* in PR China [42]. This is the first presentation of this virus in South America, although it is logical that a number of viruses such as these that solely infect arthropods are more globally widespread than currently shown.

The most prominent virus identified, found to be actively infecting 7 of the 32 collecting mosquitoes, was the *Culex* flavivirus (CxFV). This was first isolated in [43] and has since been identified in Brazil [43]. This flavivirus is not shown to infect or be harmful to humans, and it is possible that CxFV could cause mosquitoes to be less susceptible to other flaviviruses [44]. This genus includes harmful arboviruses, such as yellow fever, West Nile and Zika, that are a major concern for public health [2]. Further investigation of the inter-relations of these viruses using metagenomic approaches can help further elucidate the effects of flavivirus infection on limiting susceptibility to similar pathogens.

Of the identified viruses from this study, Shamonda orthobunyavirus is the only one identified to infect humans [45]. More generally, orthobunyaviruses infect humans globally, and are one of the most common arboviruses that infect humans in Brazil [46]. Given the nonspecific febrile symptoms associated with a litany of arboviruses, misdiagnosis of this and other lesser-known species as more well-known flaviviruses can occur. This protocol and other metagenomic assays can allow for the specific identification of arboviruses, which can be a critical tool when potentially dealing with the co-circulation of multiple pathogens at a given time [2].

### The advantage of identifying sylvatic arbovirus infections

While this proof of concept has been tested in the controlled environment of a zoo, with easily confirmable blood meal animals, and known viruses, the true potential of this assay is in its use to identify arbovirus–vector–host interactions in the wild. Using metagenomic methods for viral sequencing allows for the potential identification of novel, as yet uncharacterized viruses [19, 47]. Some of these have been identified recently in Latin America, such as the Brazilian mammarenavirus [17], although it was only possible to sequence this particular virus from heavily symptomatic cases that led to fatalities. A potential hope is that early identification of these pathogens in the future can allow for more pre-emptive treatments, lowering the overall risk of severe illness posed by an ever-increasing number of arboviruses [2, 17].

Some of the sylvatic environments that harbour these unknown arboviruses will be host to a number of unclassified species of birds and mammals, so using a global database can allow for an idea of similar species [26]. This helps identify other animals more likely to be infected by particular viruses and mosquitoes. As expected, a general observation across all of the blood meals and mosquitoes sequenced is that species of the genus *Culex* feed predominantly on birds [37]. If pathogenic viruses identified in wild populations proximal to avian farms or factories infect the animals there in a more confined space, the economic and public health risks will be significant [3, 4]. While the majority of species identified in this study belong to the genus *Culex*, it has been possible to identify other Culicidae genera that contain mosquitoes associated with numerous epidemics.

Some mosquito species, such as *Aedes aegypti* and *Haemagogus leucocelaenus*, are well classified due to the risk they pose to public health in this regard, although others have been shown to harbour pathogenic arboviruses and transfer them to human populations [2, 48]. Therefore, being able to potentially identify new species using DNA barcoding and data polishing can allow for the identification of other potentially high-risk species, when validated subsequently using morphological taxonomy. This provides a potentially large-scale method to increase the limited number of mosquito species identified on BOLD for particular regions. Moreover, these identified H–V–P interactions can eventually be applied to larger scale collaborative models that can help predict potential spillover events from sylvatic environments to urban ones.

### Differences in morphological and phylogenetic taxonomy

A potentially difficult notion is separating the differences between morphological identification and sequencing-based approaches for mosquito taxonomy. Once collected, mosquitoes were identified by taxonomic specialists to as deep a phylogenetic level as possible. For genera such as Culex, over 700 species have been identified and there are probably more that have yet to be classified [49, 50]. Moreover, identification by this method requires considerable specific training and observation skills that are not easily replicated, and delicate specimens can easily be damaged during sample collection, hindering this process. By using barcoding and other sequencing methods as complementary tools in combination with morphological analyses, it is possible to improve identification, especially for higher risk species. Moreover, conserved sequences such as COI can give a better understanding of within-species dynamics. Coupled with blood meal and viral analysis, it can be possible to identify these H–V–P interactions at a subspecies level.

### Blood pool and wider applications

Blood meal analysis is possible with two pairs of simple primers for highly conserved regions. It is possible to identify blood from birds and mammals, as well as less thought of vector groups in reptiles and amphibians. Blood meal analysis is time limited, as it will degrade over time as the mosquito breaks down and digests the blood [51]. Despite this, it is possible to amplify the partially degraded DNA for COI. Given this and based on some preliminary experiments, it is likely that after 54 h the blood meal species is no longer identifiable [52]. Taking the taxonomic hit with the most reads and binning all unmapped reads for that hit, mapping the remaining reads against the database will show whether the same reads matched to multiple species, or whether there are multiple blood sources for an individual mosquito. This appears to be the case for sample number B2359, where the mosquito was most similar to the species *C. nigripalpus*, and the majority of the blood came from reads that mapped to the Chilean flamingo. Once these reads were omitted, there were still over 1000 reads mapped to a species of black vulture. While no identifiable viruses were found for this mosquito, this can improve the speed of identifying other possible pathogen vectors for a given virus.

Additionally, this can be applied to collection traps that will sit for 1 to 3 days to collect a pool of mosquitoes, meaning individual identification is not possible. By looking at each of these on a per-read basis, it is possible to obtain an idea of the range of mosquito species present in an area, the animals they have fed on and possible viruses that are present. Whilst specific H–V–P dynamics are limited, and it is not possible to show arbovirus infection, this can provide a larger scale version of this current protocol. This also accounts for a potential limitation of this protocol in that on an individual mosquito basis the probability of finding an arbovirus of interest such as DENV or ZIKV can be quite low.

### Sample limitations

With any metagenomic, untargeted approach to sequencing, the main limiting factor will inevitably be a lack of depth of coverage. A number of decisions were made for this experiment in order to account for this. Metagenomic sequencing was only performed using a portion of each mosquito’s head to limit the number of host reads and to act as a marker for infection by ignoring viruses only found in the gut that may not infect the mosquito. In only identifying active arbovirus infections there can be much more confidence in determining whether certain viruses could then go on to infect a separate species. This may exclude viruses that would only be found in sufficient concentrations in the abdomen and would not account for the full spectrum of pathogens, but the primary intention of this assay is to focus on arboviruses more clearly infecting the mosquito at the point of capture. Only mosquitoes identified as female were used, given that males pose no epidemiological risk, thus allowing for up to 10 female mosquitoes being sequenced per flow cell. While it is possible to sequence greater or smaller numbers, this struck a balance between being cost-effective and allowing sufficient sequencing depth for each sample to identify arboviruses. Defining what determines a present arbovirus can also not be completely clear. Kraken 2 outputs show species with only one to five reads, which may correspond to infections that had subsided by the time that the mosquito was captured. As metagenomic tools for deeper, higher-throughput sequencing become more available, the ability to define pathogen infections by the method will improve. Additional aims for this protocol are to apply it to a wider array of sample types and locations, as well as implementing additional steps to reduce the amount of non-viral reads for metagenomic sequencing. These applications and potential upscaling of the protocol will be limited by the current cost of sequencing, although this has decreased significantly in the past decade, and as the technology improves metagenomic assays such as this and the original SMART-9N protocol can become more feasible as surveillance tools over time [19].

In addition to untargeted approaches using metagenomics, as in this method, more targeted approaches such as arboviral panels should be considered in future surveillance. These would not be as limited by pathogens with low viraemia and would provide a lower cost high-throughput means of surveillance once optimized. However, metagenomic approaches such as this should still be considered, given that the costs of next-generation sequencing are becoming less prohibitive, and that metagenomics potentially allows for the identification of novel viruses beyond the scope of panels, allowing for them to be optimized further.

## Conclusions

This paper presents an innovative approach for high-throughput sequencing of mosquitoes, blood meal and viruses using nanopore chemistry. This has been achieved with a combined approach of viral RNA metagenomics and DNA barcoding of COI. Using this, it has been possible to identify arboviruses and the species of mosquito they can naturally infect. Identifying recently fed-on animals in tandem with this allow for the modelling of host–vector–pathogen dynamics, identifying other species at risk of infection. More broadly speaking, this assay provides a means of directly studying these complex interactions in sylvatic environments prior to pathogens impacting on urban areas. In the face of continual urbanization, deforestation and climate change, this methodology holds potential in predicting and anticipating proximal arboviral epidemics at early stages. By shedding light on the intricate relationships between mosquitoes, hosts and viruses, this research contributes to a deeper understanding of disease transmission dynamics and facilitates more effective surveillance and intervention strategies for known and potentially novel causes of future epidemics.

## Supplementary Data

Supplementary material 1
