# Supplementary material for: Tracking arboviruses, their transmission vectors and potential hosts by nanopore sequencing of mosquitoes
Source: Microb Genom. 2024 Jan 19;10(1):001184. doi: 10.1099/mgen.0.001184 (PMC10868619; doi:10.1099/mgen.0.001184)
Supplement: Supplementary material 1 [file mgen-10-1184-s001.pdf]

Supplementary Tables

	Guimares et al., 2021					Current study									
Mosquito code	Mosquito	Bloodmeal 1	Common name	Bloodmeal 2	Common name	Result Mosquito BOLD	Results Racon and BLAST	Result Bloodmeal BOLD	Common Name	Results Bloodmeal 2 BOLD	Common Name	Barcode (Mosquito)	Barcode (Bloodmeal)	Accession IDs (Mosquito)	Accession IDs (Bloodmeal)
MZB1	*Culex declarator*	*Pipile jacutinga*	Guan (bird)	*Homo sapiens*	Human	*Culex declarator/bidens*	*Culex declarator*	*Pipile jacutinga*	Guan (bird)	*Penelope sp.*	Guan (bird)	1	33	ERX11591481	ERX11591482
MZB2	*Culex declarator*	No amplification	No amplification			*Culex declarator/bidens*	*Culex declarator*	No amplification	No amplification			2	34	ERX11591483	ERX11591484
MZB3	*Culex declarator*	*Homo sapiens*	Human			*Culex declarator/bidens*	*Culex declarator*	*Homo sapiens*	Human			3	35	ERX11591485	ERX11591486
MZB4	*Culex declarator*	*Spizaetus ornatos*	Ornate Hawk Eagle			*Culex declarator/bidens*	*Culex declarator*	*Spizaetus ornatus*	Ornate Hawk Eagle			4	36	ERX11591487	ERX11591488
MZB5	*Culex declarator*	*Nycticorax nycticorax*	Night heron			*Culex declarator/bidens*	*Culex declarator*	*Nycticorax spp.*	Night heron			5	37	ERX11591489	ERX11591490
MZB6	*Culex chidesteri*	*Homo sapiens*	Human			*Culex chidesteri*	*Culex chidesteri*	*Homo sapiens*	Human			6	38	ERX11591491	ERX11591492
MZB61	*Aedes scapularis*	*Homo sapiens*	Human			*Aedes scapularis*	*Aedes scapularis*	*Homo sapiens*	Human			7	39	ERX11591493	ERX11591494
MZB120	*Mansonia titillans*	*Homo sapiens*	Human			*Mansonia titillans*	*Mansonia titillans*	*Homo sapiens*	Human			8	40	ERX11591495	ERX11591496
MZB333	*Culex declarator*	No amplification	No amplification			*Culex declarator/bidens*	*Culex declarator*	*Ardea spp.*	Great Heron			9	41	ERX11591497	ERX11591498
MZB337	*Culex declarator*	*Cathartes melambrotus*	Turkey vulture	*Homo sapiens*	Human	*Culex declarator/bidens*	*Culex declarator*	*Cathartes spp.*	Turkey vulture	*Homo sapiens*	Human	10	42	ERX11591499	ERX11591500
MZB338	*Culex declarator*	*Canis lupus familiaris*	Domestic dog			*Culex declarator/bidens*	*Culex declarator*	No amplification	No amplification			11	43	ERX11591501	ERX11591502
MZB339	*Culex chidesteri*	No amplification	No amplification			*Culex chidesteri*	*Culex chidesteri*	No amplification	No amplification			12	44	ERX11591503	ERX11591504
MZB342	*Culex declarator*	No amplification	No amplification			*Culex declarator/bidens*	*Culex declarator*	No amplification	No amplification			13	45	ERX11591505	ERX11591506
MZB345	*Culex sp.*	*Homo sapiens*	Human			*Culex nigripalpus/declarator/bidens*	*Culex nigripalpus*	*Homo sapiens*	Human			14	46	ERX11591507	ERX11591508
MZB431	*Culex ameliae*	*Cygnus atratus*	Swan			*Culex nigripalpus/declarator/bidens*	*Culex nigripalpus*	*Cygnus spp.*	Swan			15	47	ERX11591509	ERX11591510
MZB533	*Culex Complexo Coronator*	*Cygnus atratus*	Swan	*Homo sapiens*	Human	*Culex coronator*	*Culex coronator*	*Cygnus spp.*	Swan	*Homo sapiens*	Human	16	48	ERX11591511	ERX11591512
MZB554	*Culex (Melanoconion) sp.*	No amplification	No amplification			*Culex atratus*	*Culex atratus*	No amplification	No amplification			17	49	ERX11591513	ERX11591514
MZB635	*Anopheles evansae*	*Homo sapiens*	Human			*Anopheles evansae*	*Anopheles evansae*	*Homo sapiens*	Human			18	50	ERX11591515	ERX11591516
MZB770	*Culex (Melanoconion) sp.*	No amplification	No amplification			*Culex atratus*	*Culex atratus*	No amplification	No amplification			19	51	ERX11591517	ERX11591518
MZB832	*Culex (Melanoconion) sp.*	No amplification	No amplification			*Culex atratus*	*Culex atratus*	No amplification	No amplification			20	52	ERX11591519	ERX11591520
MZB882	*Culex Complexo Coronator*	*Pavo muticus*	Peafowl			*Culex coronator*	*Culex coronator*	*Alectoris spp.*	Partridge	*Tragopan spp.*	Pheasant	21	53	ERX11591521	ERX11591522
MZB883	*Culex chidesteri*	No amplification	No amplification			*Culex chidesteri*	*Culex chidesteri*	No amplification	No amplification			22	54	ERX11591523	ERX11591524
MZB885	*Culex declarator*	*Didelphis aurita*	Opossom			*Culex declarator/bidens*	*Culex declarator*	*Didelphis spp.*	Opossom			23	55	ERX11591525	ERX11591526
MZB965	*Culex sp.*	*Homo sapiens*	Human			*Culex nigripalpus/declarator/bidens*	*Culex nigripalpus*	No amplification	No amplification			24	56	ERX11591527	ERX11591528
MZB1100	*Culex sp.*	No amplification	No amplification			*Culex nigripalpus/declarator/bidens*	*Culex nigripalpus*	No amplification	No amplification			25	57	ERX11591529	ERX11591530
MZB1110	*Culex habilitator*	*Anser anser*	Goose			*Culex habiltator*	*Culex habiltator*	No amplification	No amplification			26	58	ERX11591531	ERX11591532
MZB1226	*Culex Complexo Coronator*	*Turdus rufiventris*	True Thrush			*Culex coronator*	*Culex coronator*	*Turdus spp.*	True Thrush			27	59	ERX11591533	ERX11591534
MZB1256	*Culex sp.*	*Cygnus atratus*	Swan			*Culex nigripalpus/declarator/bidens*	*Culex nigripalpus*	*Cygnus spp.*	Swan	*Branta spp.*	Goose	28	60	ERX11591535	ERX11591536
MZB1371	*Culex sp.*	*Nycticorax nycticorax*	Night heron			*Culex nigripalpus/declarator/bidens*	*Culex nigripalpus*	*Nycticorax spp.*	Night heron			29	61	ERX11591537	ERX11591538
MZB1394	*Culex sp.*	No amplification	No amplification			*Culex usquatus*	*Culex usquatus*	No amplification	No amplification			30	62	ERX11591539	ERX11591540
MZB1823	*Culex Complexo Coronator*	*Homo sapiens*	Human			*Culex coronator*	*Culex coronator*	No amplification	No amplification			31	63	ERX11591541	ERX11591542
MZB2073	*Culex (Melanoconion) sp.*	*Ardea herodias*	Great Heron			*Culex atratus*	*Culex atratus*	*Ardea spp.*	Great Heron			32	64	ERX11591543	ERX11591544
MZB2078	*Culex (Melanoconion) sp.*	*Nycticorax nycticorax*	Night heron			*Culex atratus*	*Culex atratus*	*Nycticorax spp.*	Night heron			89	90	ERX11591545	ERX11591546

Table S1: Comparison of Guimarães et al. (36) study and results with the current work in terms of mosquito and blood meal identification for mosquitoes collected in 2015. For blood meals, both species names and common names have been used and samples with multiple blood meals present are listed in subsequent columns. Bloodmeals marked with “No amplification” were the samples where there were not a sufficient number of reads mapping to any of the reference fasta files in the BOLD database. Fastq files for each sample mosquito and bloodmeal are listed by accession ID and are accessible via the European Nucleotide Archive website.

Mosquito ID	Morphological identification	Racon and BLAST identification	Bloodmeal	Common name	Possible Origin	Most closely related species in the zoo	Collection date	Collection site	Coordinates	Collection time	Temperature (C°)	Humidity (%)	Accession IDs (Metagenomics)	Accession IDs (Mosquito)	Accession IDs (Bloodmeal)
B1613	*Culex (Cux.) chidesteri*	*Culex chidesteri*	*Bucorvus abyssinicus*	Abyssinian ground hornbill	Sao Paulo Zoo	*Bucorvus abyssinicus*	03/11/20	Flamingo Enclosure (FE)	23°38'55.2"S 46°37'16.8"W	17:10:00	18.4	54	ERX11591547	ERX11591547	ERX11591548
B1618	*Culex (Cux.) chidesteri*	*Culex chidesteri*	*Bucorvus abyssinicus*	Abyssinian ground hornbill	Sao Paulo Zoo	*Bucorvus abyssinicus*	03/11/20	Flamingo Enclosure (FE)	23°38'55.2"S 46°37'16.8"W	17:10:00	18.4	54	ERX11591549	ERX11591549	ERX11591550
B1622	*Culex (Cux.) quinquefasciatus*	*Culex quinquefasciatus*	*Phoenicopterus chilensis*	Chilean flamingo	Sao Paulo Zoo	*Phoenicopterus chilensis*	03/11/20	Flamingo Enclosure (FE)	23°38'55.2"S 46°37'16.8"W	17:10:00	18.4	54	ERX11591551	ERX11591551	ERX11591552
B1623	*Culex (Cux.) chidesteri*	*Culex chidesteri*	*Phoenicopterus chilensis*	Chilean flamingo	Sao Paulo Zoo	*Phoenicopterus chilensis*	03/11/20	Flamingo Enclosure (FE)	23°38'55.2"S 46°37'16.8"W	17:10:00	18.4	54	ERX11591553	ERX11591553	ERX11591554
B1624	*Culex (Cux.) chidesteri*	*Culex chidesteri*	*Laterallus albigularis*	White-throated crake	Wildlife	*Rallidae (familia)*	03/11/20	Flamingo Enclosure (FE)	23°38'55.2"S 46°37'16.8"W	17:10:00	18.4	54	ERX11591555	ERX11591555	ERX11591556
B1647	*Culex (Cux.) chidesteri*	*Culex chidesteri*	*Coragyps atratus*	Black vulture	Wildlife	*Coragyps atratus*	03/11/20	Flamingo Enclosure (FE)	23°38'55.2"S 46°37'16.8"W	17:10:00	18.4	54	ERX11591557	ERX11591557	ERX11591558
B1649	*Culex (Cux.) sp.*	*Culex bidens*	*Phoenicopterus chilensis*	Chilean flamingo	Sao Paulo Zoo	*Phoenicopterus chilensis*	03/11/20	Flamingo Enclosure (FE)	23°38'55.2"S 46°37'16.8"W	17:10:00	18.4	54	ERX11591559	ERX11591559	ERX11591560
B1667	*Culex (Cux.) Complexo Coronator*	*Culex coronator*	*Phoenicopterus chilensis*	Chilean flamingo	Sao Paulo Zoo	*Phoenicopterus chilensis*	03/11/20	Flamingo Enclosure (FE)	23°38'55.2"S 46°37'16.8"W	17:10:00	18.4	54	ERX11591561	ERX11591561	ERX11591562
B1668	*Culex (Cux.) sp.*	*Culex bidens*	*Phoenicopterus chilensis*	Chilean flamingo	Sao Paulo Zoo	*Phoenicopterus chilensis*	03/11/20	Flamingo Enclosure (FE)	23°38'55.2"S 46°37'16.8"W	17:10:00	18.4	54	ERX11591563	ERX11591563	ERX11591564
B1804	*Culex (Cux.) chidesteri*	*Culex chidesteri*	*Phoenicopterus chilensis*	Chilean flamingo	Sao Paulo Zoo	*Phoenicopterus chilensis*	01/12/20	West of Giraffe House (WGH)	23°38'58.3"S 46°37'03.9"W	17:07:00	25.1	87	ERX11591565	ERX11591565	ERX11591566
B1884	*Culex (Cux.) sp.*	*Culex imitator*	*Homo sapiens*	Human	Visitor or employee		01/12/20	West of Giraffe House (WGH)	23°39'01.5"S 46°37'03.0"W	17:20:00	25.1	87	ERX11591567	ERX11591567	ERX11591568
B1959	*Culex (Cux.) sp.*	*Culex aureonotatus*	*Phoenicopterus chilensis*	Chilean flamingo	Sao Paulo Zoo	*Phoenicopterus chilensis*	01/12/20	West of Giraffe House (WGH)	23°39'01.5"S 46°37'03.0"W	17:20:00	25.1	87	ERX11591569	ERX11591569	ERX11591570
B2106	*Culex (Cux.) chidesteri*	*Culex chidesteri*	*Didelphis albiventris*	White-eared opossum	Wildlife	*Didelphis aurita*	01/12/20	Collection site no.69 (R69)	23°39'11.2"S 46°36'59.7"W	17:49:00	25.1	87	ERX11591571	ERX11591571	ERX11591572
B2123	*Culex (Cux.) sp.*	*Culex nigripalpus*	*Homo sapiens*	Human	Visitor or employee		01/12/20	Collection site no.69 (R69)	23°39'11.2"S 46°36'59.7"W	17:49:00	25.1	87	ERX11591573	ERX11591573	ERX11591574
B2125	*Culex (Cux.) sp.*	*Culex nigripalpus*	*Phoenicopterus chilensis*	Chilean flamingo	Sao Paulo Zoo	*Phoenicopterus chilensis*	01/12/20	Collection site no.69 (R69)	23°39'11.2"S 46°36'59.7"W	17:49:00	25.1	87	ERX11591575	ERX11591575	ERX11591576
B2191	*Culex (Cux.) chidesteri*	*Culex chidesteri*	*Phoenicopterus chilensis*	Chilean flamingo	Sao Paulo Zoo	*Phoenicopterus chilensis*	01/12/20	Flamingo Enclosure (FE)	23°38'55.2"S 46°37'16.8"W	18:31:00	25.1	87	ERX11591577	ERX11591577	ERX11591578
B2235	*Culex (Cux.) chidesteri*	*Culex chidesteri*	*Bucorvus_abyssinicus*	Abyssinian ground hornbill	Sao Paulo Zoo	*Bucorvus abyssinicus*	01/12/20	Flamingo Enclosure (FE)	23°38'55.2"S 46°37'16.8"W	18:31:00	25.1	87	ERX11591579	ERX11591579	ERX11591580
B2298	*Culex (Cux.) sp.*	*Culex nigripalpus*	*Aramus guarau*	Limpkin	Wildlife	*Aramus guarauna*	01/12/20	Flamingo Enclosure (FE)	23°38'55.2"S 46°37'16.8"W	18:31:00	25.1	87	ERX11591581	ERX11591581	ERX11591582
B2339	*Culex (Cux.) chidesteri*	*Culex chidesteri*	*Phoenicopterus chilensis*	Chilean flamingo	Sao Paulo Zoo	*Phoenicopterus chilensis*	01/12/20	Flamingo Enclosure (FE)	23°38'55.2"S 46°37'16.8"W	18:31:00	25.1	87	ERX11591583	ERX11591583	ERX11591584
B2343	*Culex (Cux.) sp.*	*Culex nigripalpus*	*Phoenicopterus chilensis*	Chilean flamingo	Sao Paulo Zoo	*Phoenicopterus chilensis*	01/12/20	Flamingo Enclosure (FE)	23°38'55.2"S 46°37'16.8"W	18:31:00	25.1	87	ERX11591585	ERX11591585	ERX11591586
B2359	*Culex (Cux.) sp.*	*Culex nigripalpus*	*Phoenicopterus chilensis*	Chilean flamingo	Sao Paulo Zoo	*Phoenicopterus chilensis*	01/12/20	Flamingo Enclosure (FE)	23°38'55.2"S 46°37'16.8"W	18:31:00	25.1	87	ERX11591587	ERX11591587	ERX11591588
B2363	*Culex (Cux.) sp.*	*Culex nigripalpus*	*Phoenicopterus chilensis*	Chilean flamingo	Sao Paulo Zoo	*Phoenicopterus chilensis*	01/12/20	Flamingo Enclosure (FE)	23°38'55.2"S 46°37'16.8"W	18:31:00	25.1	87	ERX11591589	ERX11591589	ERX11591590
B2396	*Culex (Cux.) sp.*	*Culex nigripalpus*	*Coragyps atratus*	Black vulture	Wildlife	*Coragyps atratus*	01/12/20	Flamingo Enclosure (FE)	23°38'55.2"S 46°37'16.8"W	18:31:00	25.1	87	ERX11591591	ERX11591591	ERX11591592
B2400	*Culex (Cux.) sp.*	*Culex aureonotatus*	*Campephilus melanoleucos*	Crimson-crested woodpecker	Wildlife	*Picidae (familia)*	01/12/20	Flamingo Enclosure (FE)	23°38'55.2"S 46°37'16.8"W	18:31:00	25.1	87	ERX11591593	ERX11591593	ERX11591594
B2419	*Culex (Cux.) sp.*	*Culex aureonotatus*	*Columbi passeri*	Dove	Wildlife	*Columbidae (familia)*	01/12/20	Flamingo Enclosure (FE)	23°38'55.2"S 46°37'16.8"W	18:31:00	25.1	87	ERX11591595	ERX11591595	ERX11591596
B2445	*Culex (Mel.) sp.*	*Culex aureonotatus*	*Homo sapiens*	Human	Visitor or employee		01/12/20	Flamingo Enclosure (FE)	23°38'55.2"S 46°37'16.8"W	18:31:00	25.1	87	ERX11591597	ERX11591597	ERX11591598
B2468	*Culex (Cux.) sp.*	*Culex imitator*	*Phoenicopterus chilensis*	Chilean flamingo	Sao Paulo Zoo	*Phoenicopterus chilensis*	01/12/20	Flamingo Enclosure (FE)	23°38'55.2"S 46°37'16.8"W	18:31:00	25.1	87	ERX11591599	ERX11591599	ERX11591600
B2485	*Culex (Mel.) sp.*	*Culex bidens*	*Didelphis aurita*	Opossum	Wildlife		01/12/20	Flamingo Enclosure (FE)	23°38'55.2"S 46°37'16.8"W	18:31:00	25.1	87	ERX11591601	ERX11591601	ERX11591602
B2486	*Culex (Mel.) sp.*	*Culex bidens*	*Homo sapiens*	Human	Visitor or employee		01/12/20	Flamingo Enclosure (FE)	23°38'55.2"S 46°37'16.8"W	18:31:00	25.1	87	ERX11591603	ERX11591603	ERX11591604
B2613	*Culex (Mel.) sp.*	*Culex bidens*	*Bucorvus abyssinicus*	Abyssinian ground hornbill	Sao Paulo Zoo	*Bucorvus abyssinicus*	01/12/20	North of Science Complex (NSC)	23°38'48.2"S 46°37'14.4"W	18:48:00	25.1	87	ERX11591605	ERX11591605	ERX11591606
B2638	*Culex (Cux.) chidesteri*	*Culex chidesteri*	*Phoenicopterus chilensis*	Chilean flamingo	Sao Paulo Zoo	*Phoenicopterus chilensis*	01/12/20	North of Science Complex (NSC)	23°38'48.2"S 46°37'14.4"W	18:48:00	25.1	87	ERX11591607	ERX11591607	ERX11591608
B2673	*Culex (Mel.) sp.*	*Culex bidens*	*Phoenicopterus chilensis*	Chilean flamingo	Sao Paulo Zoo	*Phoenicopterus chilensis*	01/12/20	North of Science Complex (NSC)	23°38'48.2"S 46°37'14.4"W	18:48:00	25.1	87	ERX11591609	ERX11591609	ERX11591610

Table S2: Mosquito ID codes and relevant data corresponding to their taxonomic identification with morphology as well as the identified mosquito species, blood meal, and any viruses present as illustrated in Figure 5. Relevant metadata in terms of collection location, time, date, and closely related species identified in PEFI, is also described per sample ID. Fastq files for each sample are listed by accession ID and are accessible via the European Nucleotide Archive website.

Supplementary Table legend

Table S1: Comparison of Guimarães et al.(37) study and results with the current work in terms of mosquito and blood meal identification for mosquitoes collected in 2015. For blood meals, both species names and common names have been used and samples with multiple blood meals present are listed in subsequent columns. Bloodmeals marked with "No amplification" were the samples where there were not a sufficient number of reads mapping to any of the reference fasta files in the BOLD database. Fastq files for each sample mosquito and bloodmeal are listed by accession ID and are accessible via the European Nucleotide Archive website.

Table S2: Mosquito ID codes and relevant data corresponding to their taxonomic identification with morphology as well as the identified mosquito species, blood meal, and any viruses present as illustrated in Figure 5. Relevant metadata in terms of collection location, time, date, and closely related species identified in PEFI, is also described per sample ID. Fastq files for each sample are listed by accession ID and are accessible via the European Nucleotide Archive website.
